# Supplementary material for: Hyperbranched polymer functionalized flexible perovskite solar cells with mechanical robustness and reduced lead leakage
Source: Nat Commun. 2023 Oct 13;14:6451. doi: 10.1038/s41467-023-41931-1 (PMC10576085; doi:10.1038/s41467-023-41931-1)
Supplement: Supplementary file 1 — Supplementary Information [file 41467_2023_41931_MOESM1_ESM.pdf]

SUPPLEMENTARY INFORMATION

Hyperbranched polymer functionalized flexible perovskite solar cells with mechanical robustness and reduced lead leakage

Zhihao Li[1, 2, 3, 4], Chunmei Jia[1], Zhi Wan[1], Jiayi Xue[1], Junchao Cao[2, 3], Meng Zhang[1, 2], Can Li[1, 4], Jianghua Shen[2, 3], Chao Zhang[2, 3*], Zhen Li[1, 4*]

1. State Key Laboratory of Solidification Processing, Center for Nano Energy Materials, School of Materials Science and Engineering, Northwestern Polytechnical University and Shaanxi Joint Laboratory of Graphene (NPU), Xi'an, 710072, P. R. China

2. Department of Aeronautical Structure Engineering, School of Aeronautics, Northwestern Polytechnical University, Xi'an, Shaanxi 710072, China

3. Shaanxi Key Laboratory of Impact Dynamics and Its Engineering Application, Joint International Research Laboratory of Impact Dynamics and Its Engineering Applications, Xi'an, Shaanxi 710072, China

4. Research & Development Institute of Northwestern Polytechnical University in Shenzhen, Shenzhen 519057, China

E-mail: chaozhang@nwpu.edu.cn, lizhen@nwpu.edu.cn

This PDF file includes:

Supplementary Figures 1 to 48

Supplementary Table 1 to 8

Supplementary Note 1 to 3

Supplementary References 1 to 5

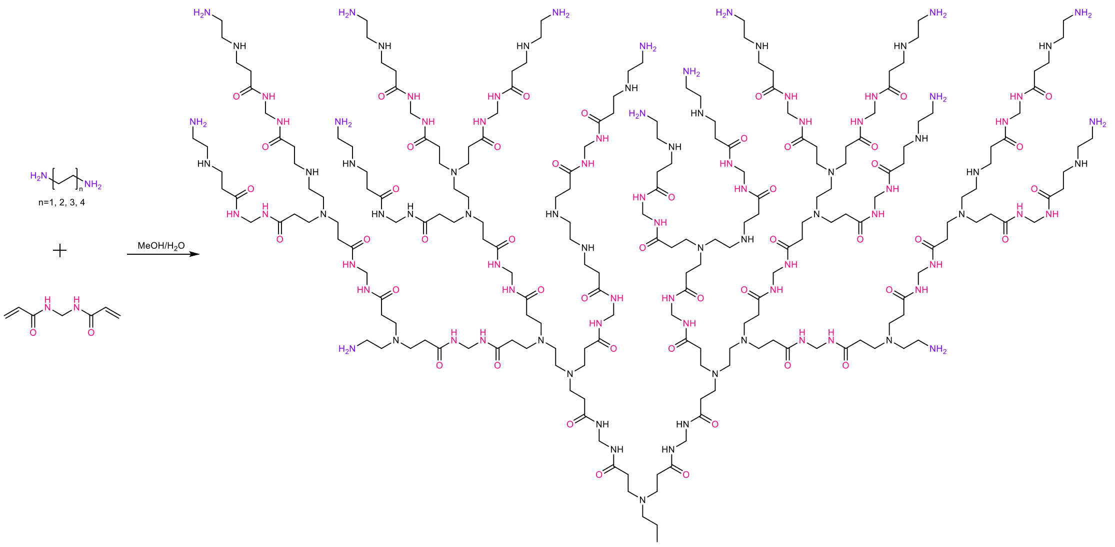

Supplementary Figure 1. Synthesis route of a series of polyamide-amine-based Hyperbranched Polymers (HBPs).

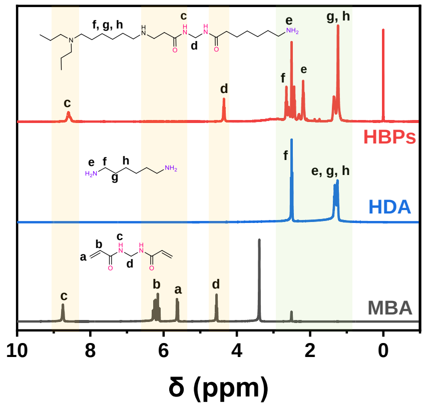

Supplementary Figure 2. ^{1}H-NMR spectroscopy of the HDA-HBPs and dimethyl sulfoxide-d_6 (DMSO) as solvent.

The typical proton signals at 6.2 and 5.6 ppm corresponding to the double bond peaks of vinyl groups disappeared after reaction, which indicated that all the vinyl groups were completely reacted and consumed. Meanwhile, the $-CH_2-NH-$ group at 8.8 and 4.5 ppm of MBA as well as the methylene group at 1.1 and 2.5 ppm of HDA were all observed in HDA-HBPs, indicating that the hyperbranched polymer was successfully prepared.

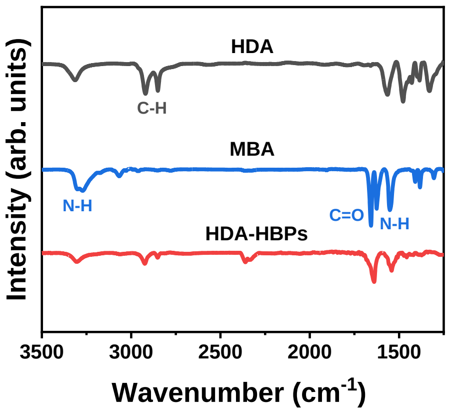

Supplementary Figure 3. FT-IR spectrum of the HDA-HBPs.

The characteristic bands at 3295, 1659, and 1550 cm^{-1} belong to the strong stretching vibration of N-H group, C=O stretching vibration band and N-H bending vibration band in MBA, respectively. The characteristic peaks at 2925 and 2854 cm^{-1} correspond to C-H vibrations in the HDA, indicating that the hyperbranched polymer is successfully prepared. Moreover, in the FTIR spectrum of HDA-HBPs, the characteristic amide I and amide II bands shift to 1638 and 1543 cm^{-1}, respectively, which are attributed to the formation of the hydrogen bonds.

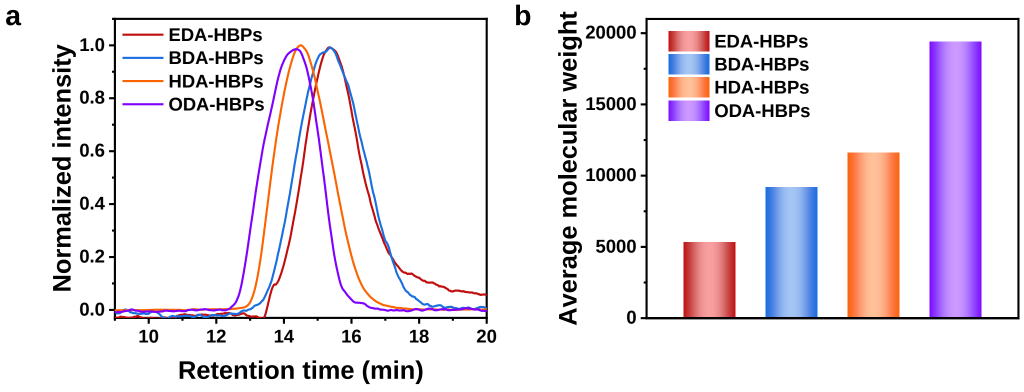

Supplementary Figure 4. (a) GPC curves and (b) average molecular wight of a series of HBPs.

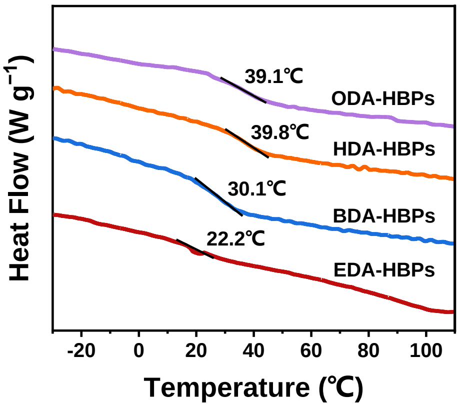

Supplementary Figure 5. Differential scanning calorimetry (DSC) curves of various HBPs

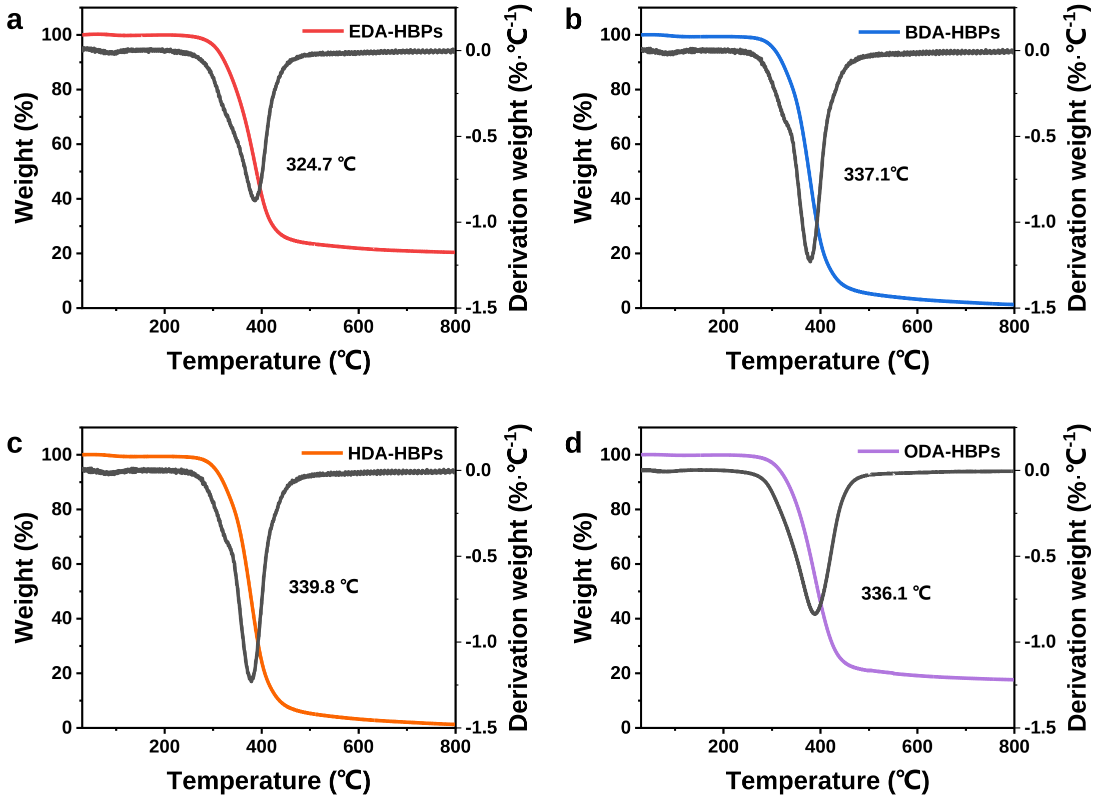

Supplementary Figure 6. Thermogravimetric analysis (TGA) curves of HBPs with (a) EDA, (b) BDA, (c) HDA and (d) ODA.

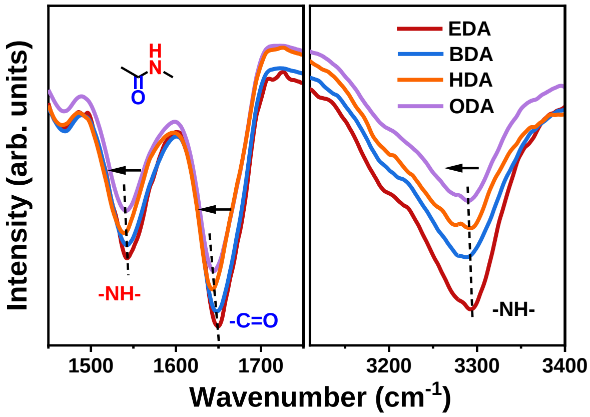

Supplementary Figure 7. FT-IR spectrum of various HBPs with different chain lengths.

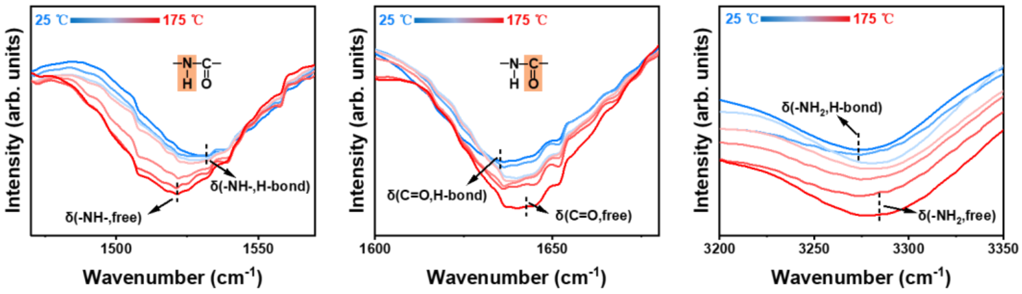

Supplementary Figure 8. Changes of hydrogen bonds in HBPs at raising temperature: temperature-dependent FTIR spectra of HBPs upon heating from 25 to 175℃, spectra at 25, 50, 75,100, 125, 150 and 175 ℃ are presented with color from blue to red.

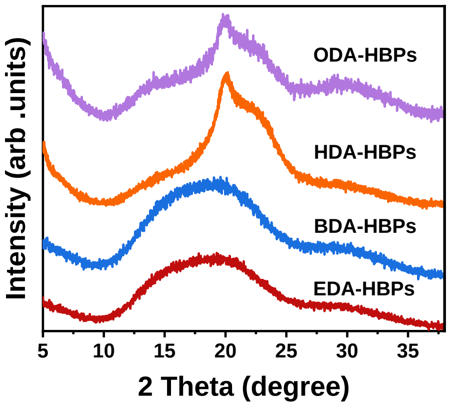

Supplementary Figure 9. XRD spectra of a series of HBPs with different chain lengths.

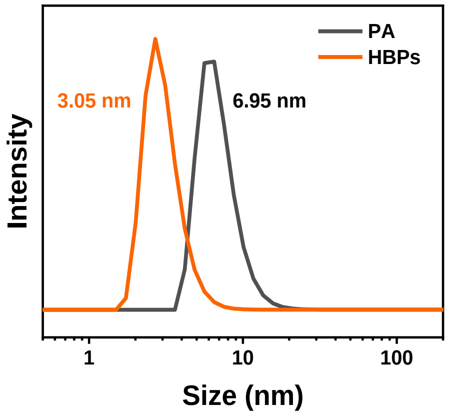

Supplementary Figure 10. Particle size distribution of PA and HBPs in methanol solution.

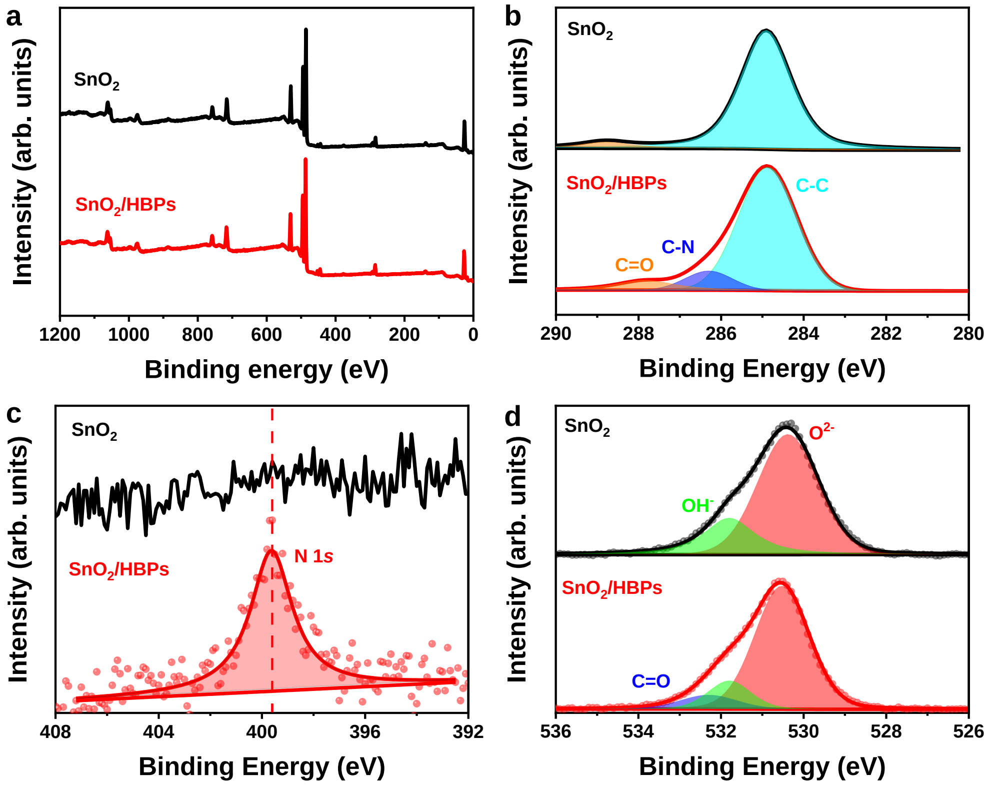

Supplementary Figure 11. X-ray photoelectron spectra (XPS) of (a) Full XPS, (b) C, (c) N and (d) O of pristine SnO_2 and HBPs-modified SnO_2 films.

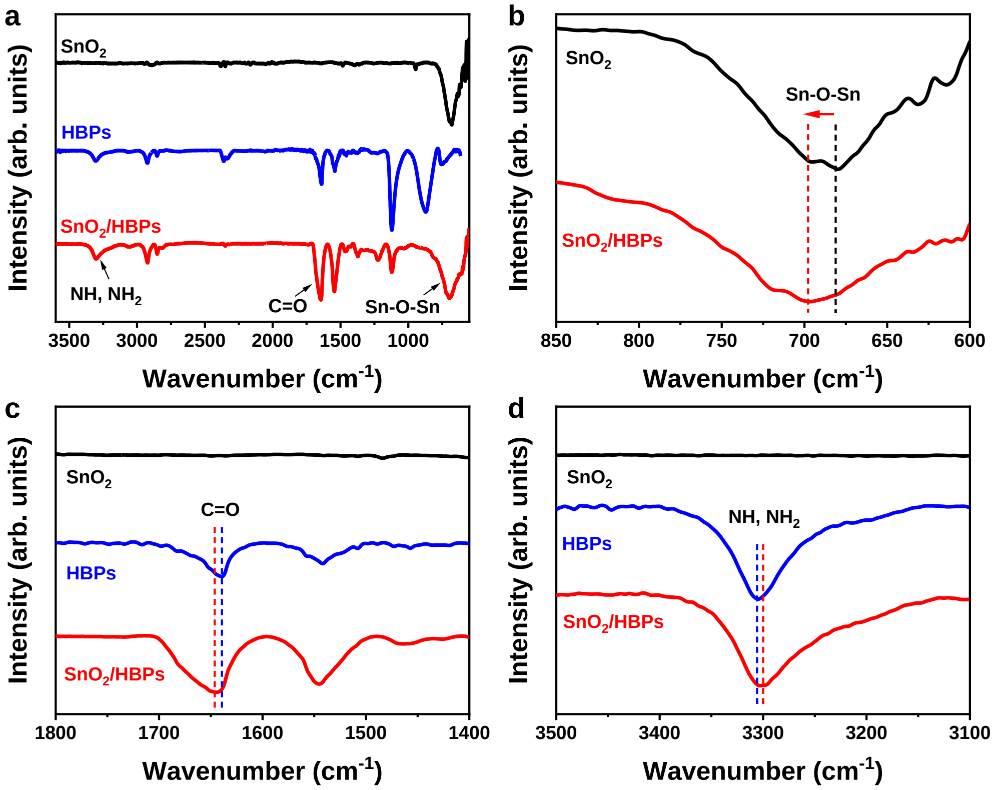

Supplementary Figure 12. Fourier-transform infrared (FTIR) spectra of (a) Full, (b) Sn-O-Sn, (c) C=O and (d) NH, NH_2 of pristine SnO_2 and HBPs-modified SnO_2 films.

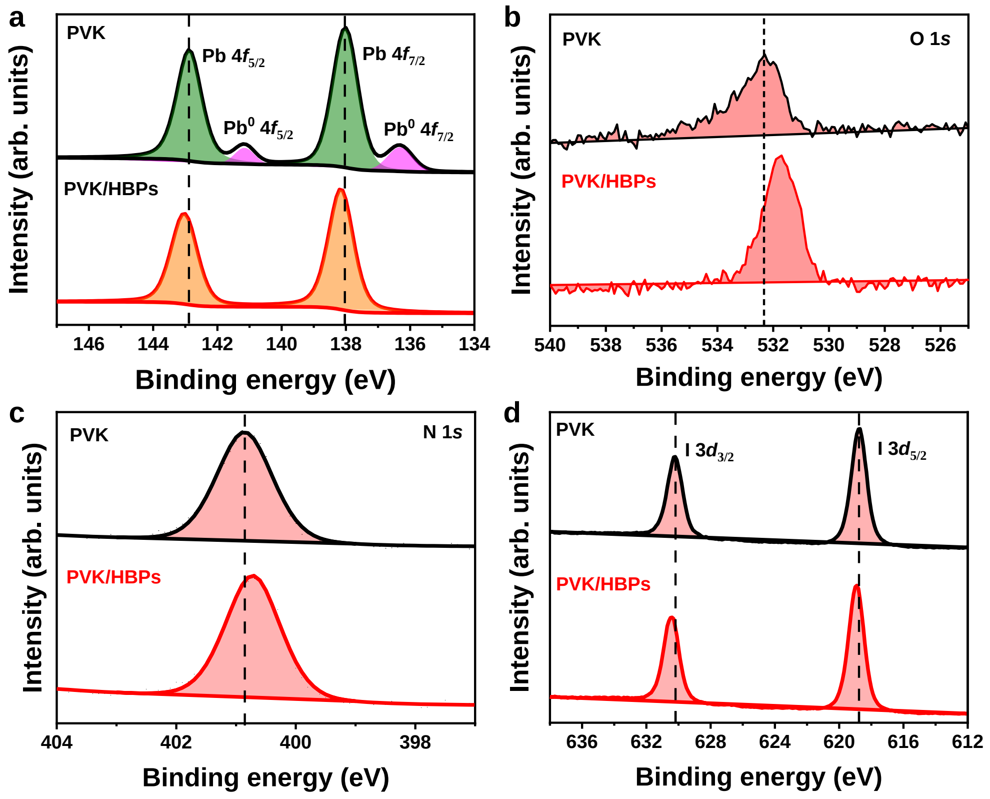

Supplementary Figure 13. X-ray photoelectron spectra (XPS) of (a) Pb, (b) O, (c) N and (d) I of pristine PVK and HBPs-modified PVK films.

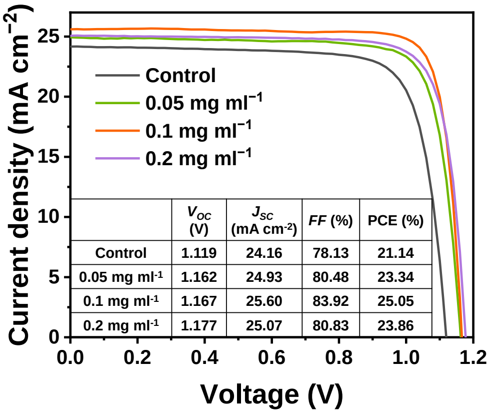

Supplementary Figure 14. Optimum *J-V* curves of the PSCs with different modification concentration (mg ml^{-1}) of HBPs in the reverse scan direction.

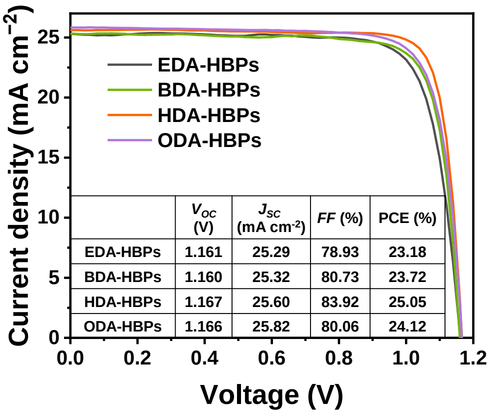

Supplementary Figure 15. Optimum *J-V* curves of the PSCs with different HBPs in the reverse scan direction.

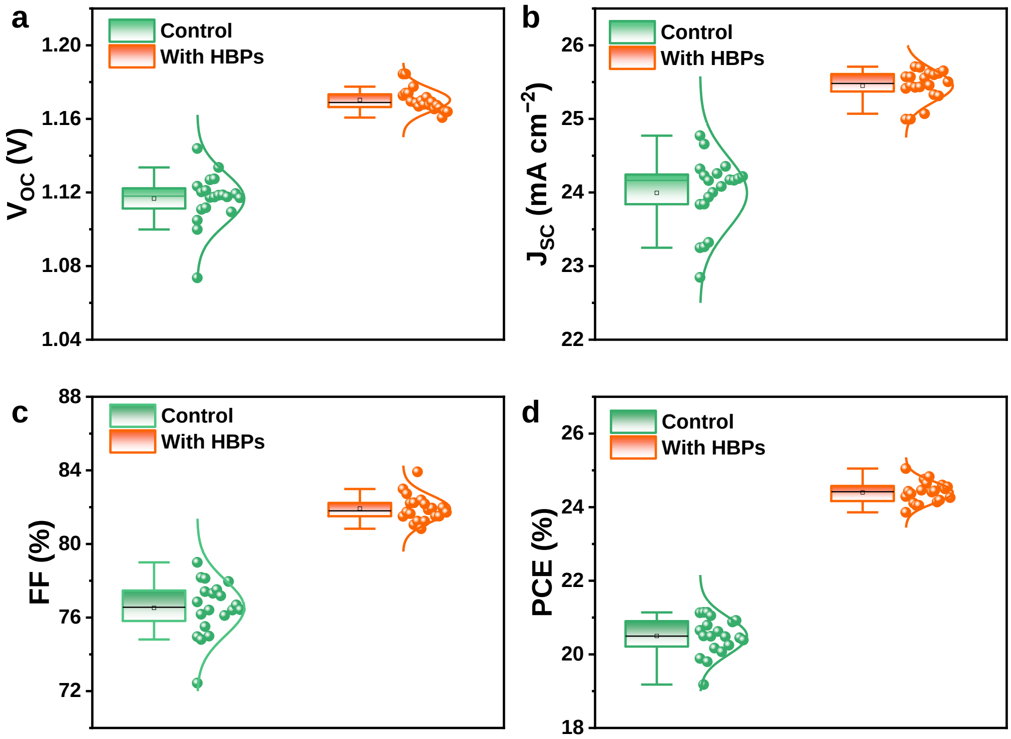

Supplementary Figure 16. (a) V_{OC}, (b) J_{SC}, (c) FF, and (d) PCE statistics of 20 devices for the control and HBPs-modified PSCs. Error bars represent the standard deviations from the statistic results of two devices.

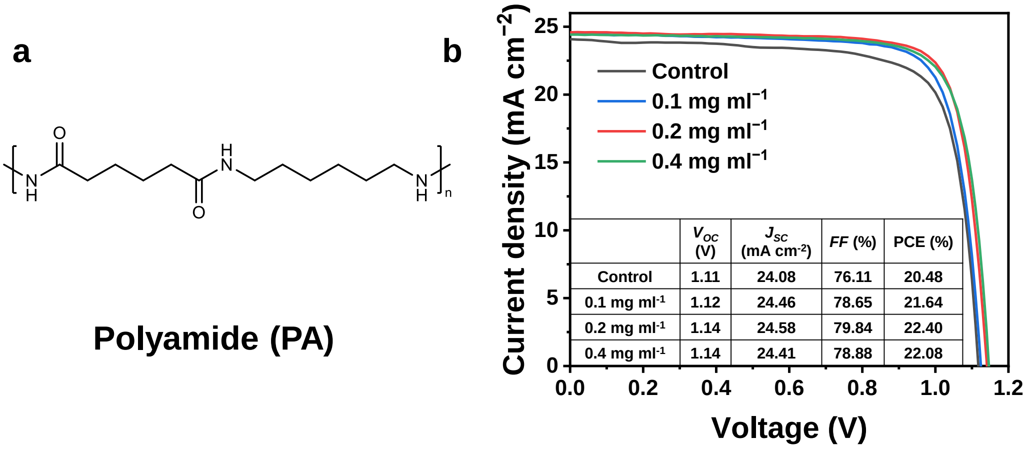

Supplementary Figure 17. (a) Chemical structure of PA polymer. (b) Optimum *J-V* curves of the PSCs with different PA polymer concentration in the reverse scan direction.

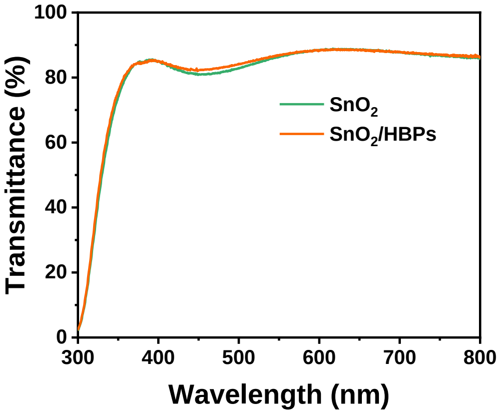

Supplementary Figure 18. Transmission spectra of the of pristine SnO_2 and the HBPs-modified SnO_2 films on the ITO substrates.

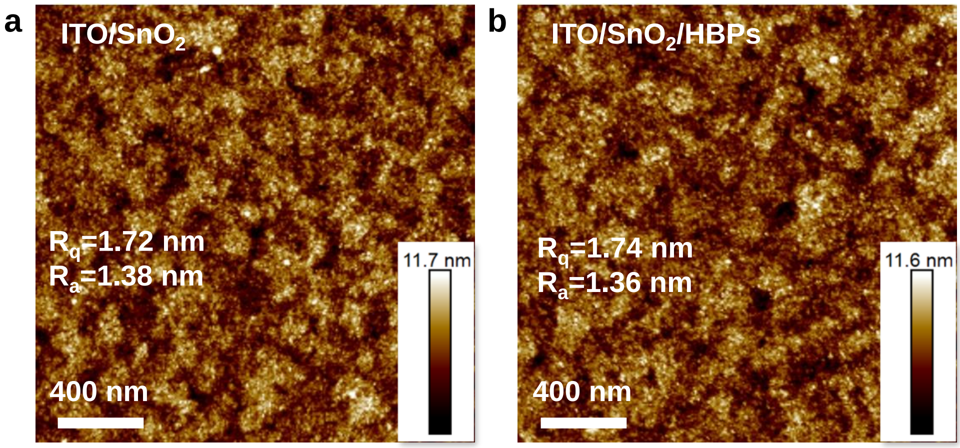

Supplementary Figure 19. AFM images of (a) Pristine-SnO_2 and (b) HBPs-modified SnO_2 films on the ITO substrates.

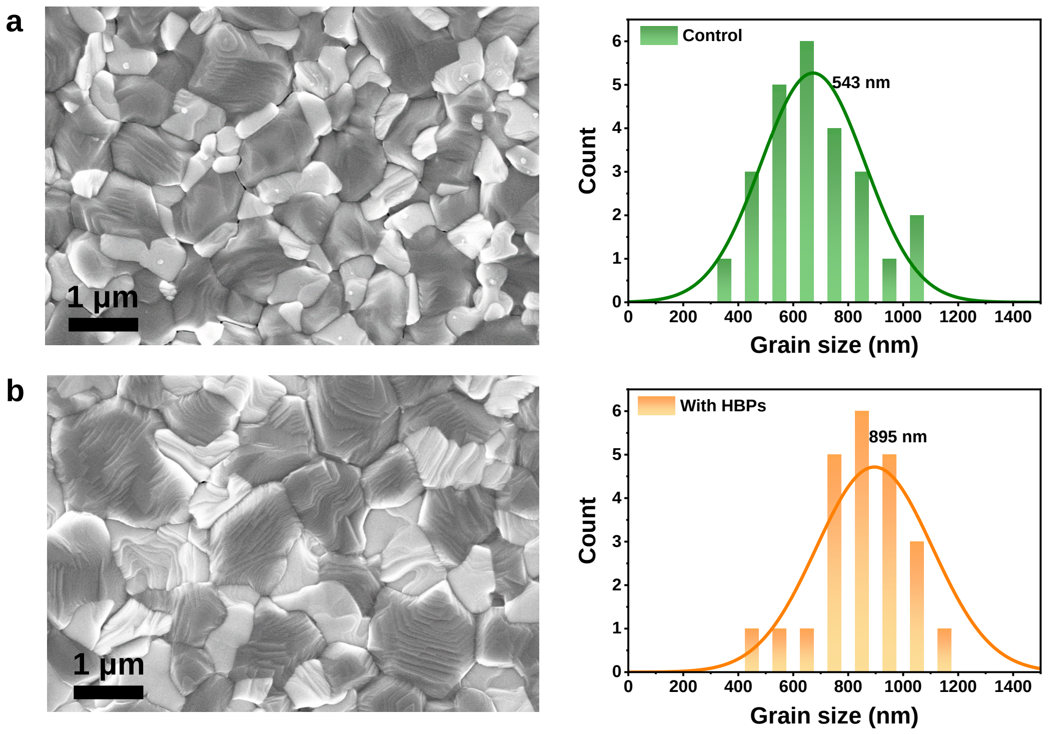

Supplementary Figure 20. Top-view scanning electron microscopy (SEM) images and corresponding grain size statistical distribution of the perovskite films based on (a) pristine SnO_2 and (b) HBPs-modified SnO_2 ETL.

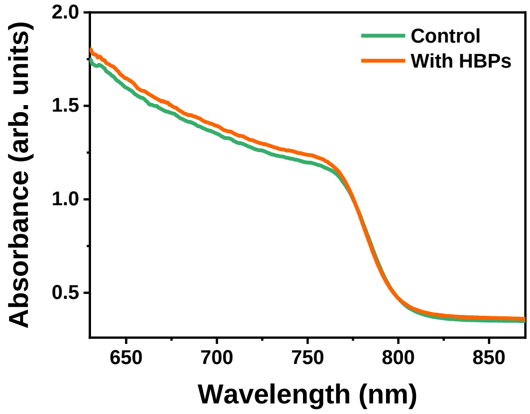

Supplementary Figure 21. UV-Vis absorption spectra of the perovskite films based on pristine SnO_2 and HBPs-modified SnO_2.

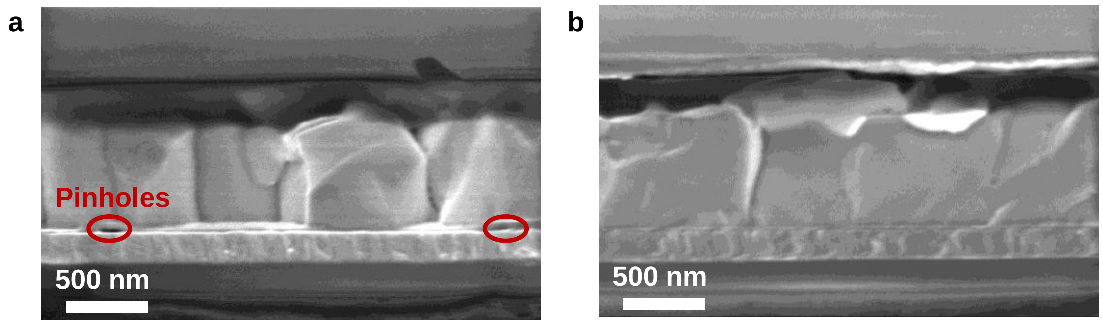

Supplementary Figure 22. Cross-sectional SEM images of the perovskite films based on (a) SnO_2 and (b) HBPs-modified SnO_2. Red circled areas are macroscopic defects (pinholes).

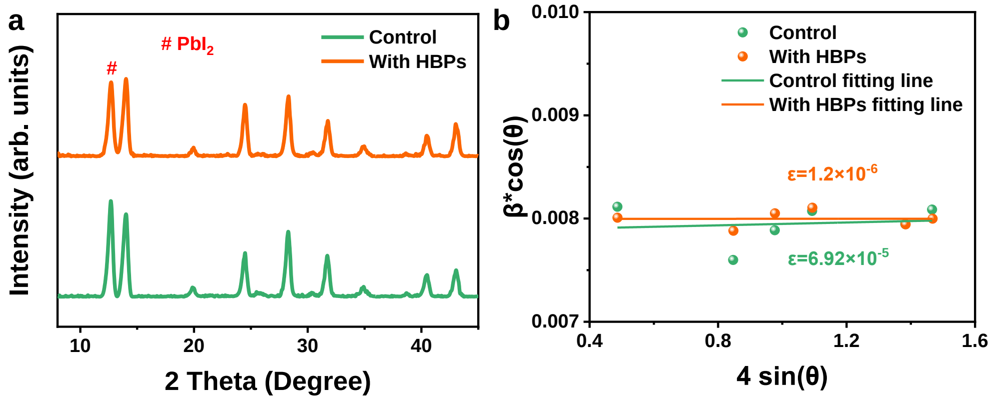

Supplementary Figure 23. X-ray diffraction (XRD) patterns (a) and calculation of micro-strains (b) in perovskite films based on SnO_2 with and without HBPs modification.

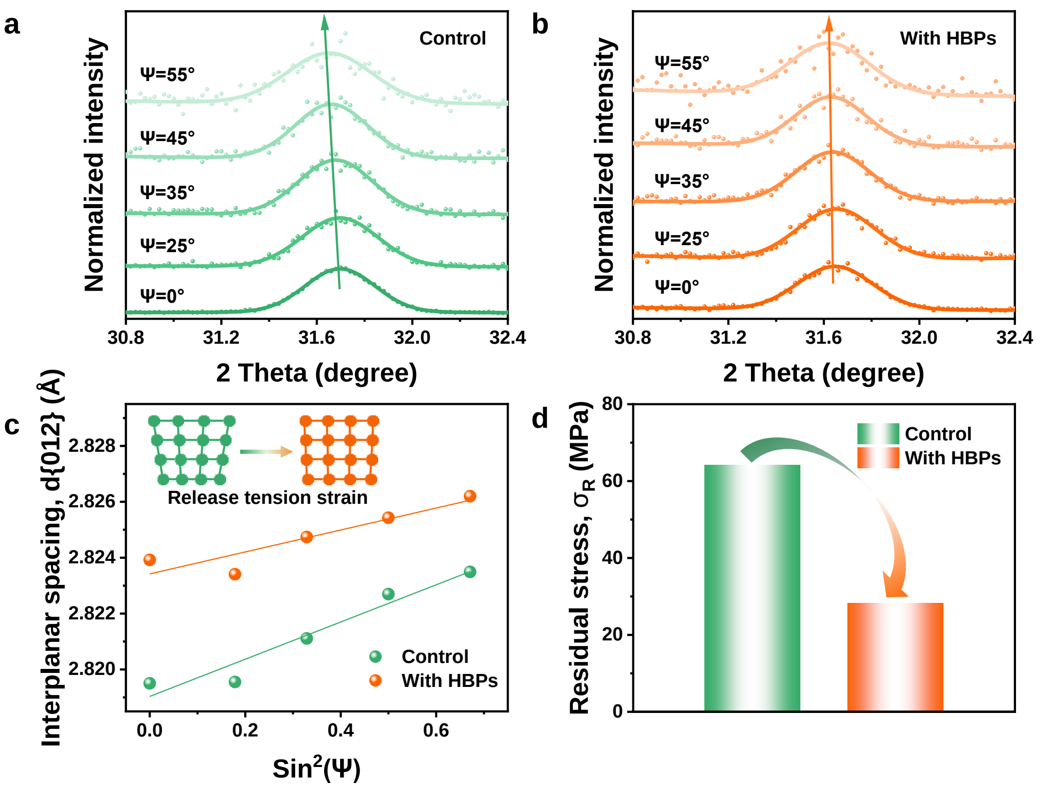

Supplementary Figure 24. Residual stress of perovskite films: GIXRD patterns at different Ψ angles (from 0° to 55°) for perovskite films formed on SnO_2 ETLs (a) control and (b) with HBPs modification, respectively. (c) Lattice spacing $d_{(012)}$ versus $sin^2(\Psi)$ plots for perovskite films formed on SnO_2 ETLs without and with HBPs, respectively. (d) Residual stress σ_R comparison between control and HBPs-modified perovskite films.

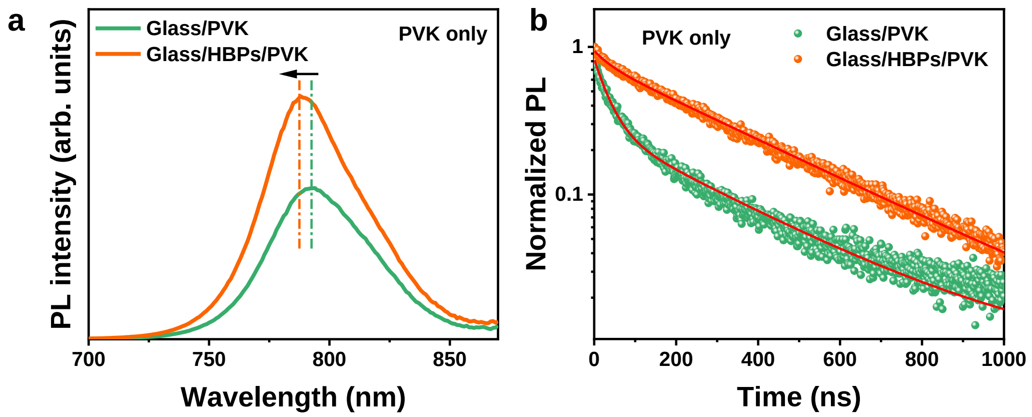

Supplementary Figure 25. (a) Photoluminescence and (b) time-resolved photoluminescence spectra of perovskite films deposited on glass with and without

HBPs modification.

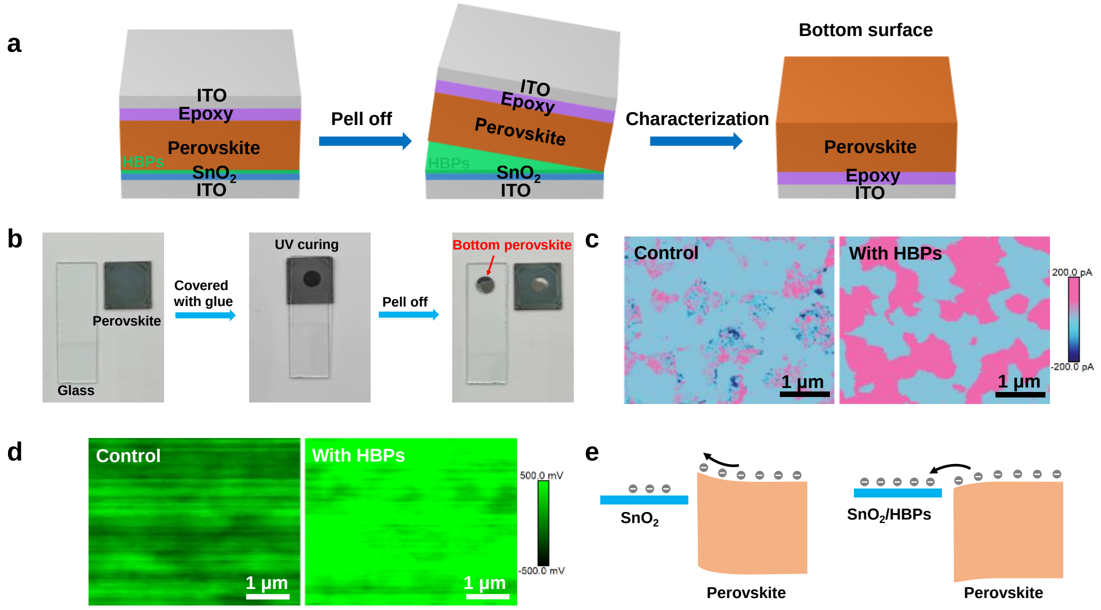

Supplementary Figure 26. (a) Schematic of process to expose the buried interface and (b) corresponding sample photos. (c) Conductive atomic force microscopy images of the buried interface in control and target devices. (d) Surface potential distribution of the buried interface in control and target device measured by Kelvin probe force microscopy; (e) Schematic diagram of control and target buried interface

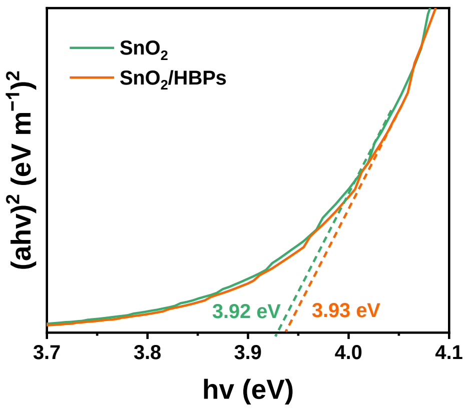

Supplementary Figure 27. Tauc plots of Pristine-SnO_2 and HBPs-modified SnO_2 films.

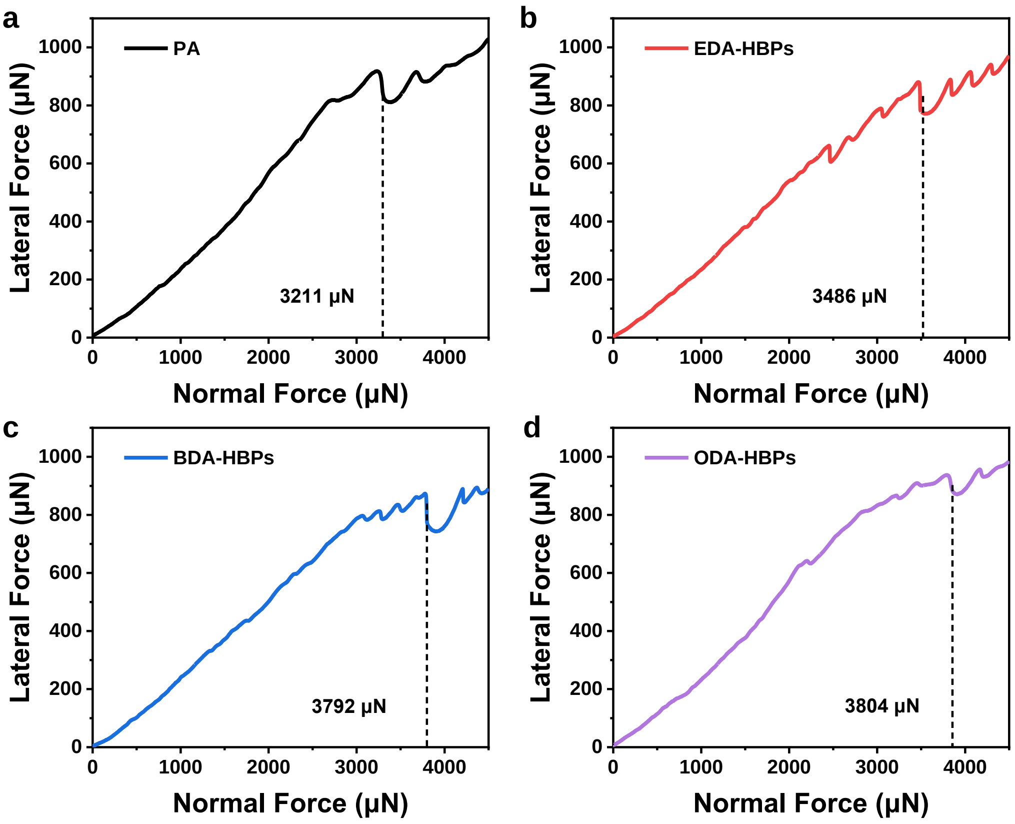

Supplementary Figure 28. The scratch force curve of perovskite film based on SnO_2 modified by (a) PA, (b) EDA-HBPs, (c) BDA-HBPs and (d) ODA-HBPs.

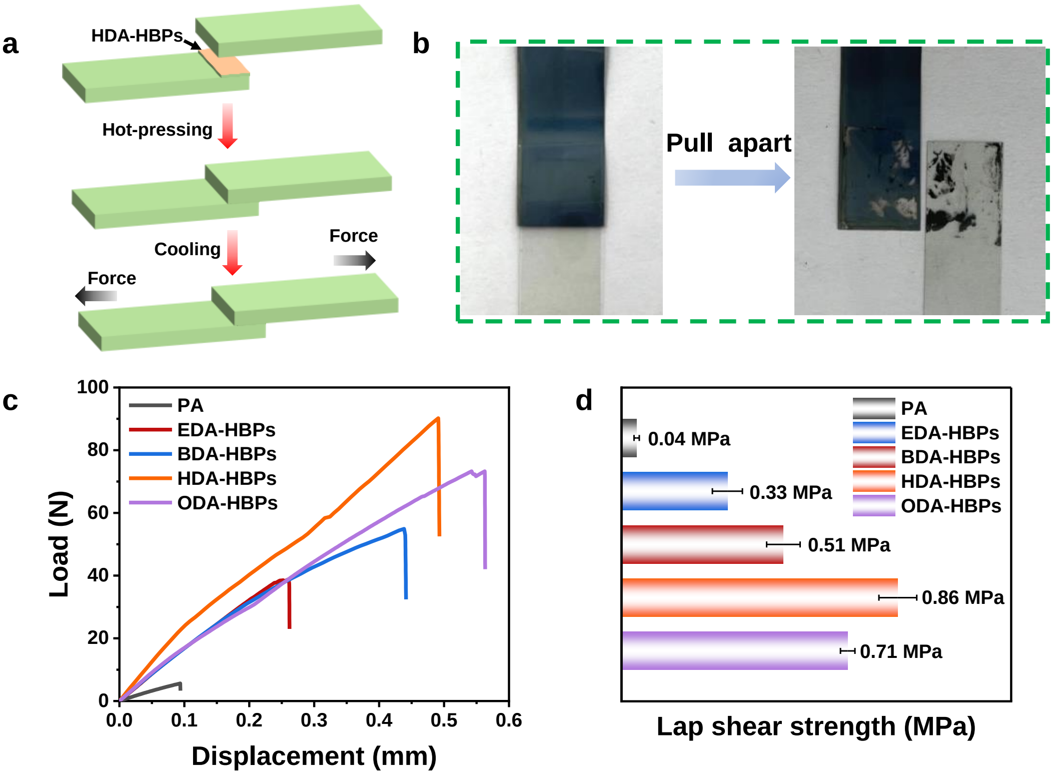

Supplementary Figure 29. (a) Schematic diagram of the adhesive procedure. (b)

Photographs of HBPs glued device after the lap shear testing with a front cell of ITO/SnO_2/PVK and a back cell of ITO/SnO_2 (HBPs). (c) Lap shear curve and (d) adhesion strengths of different polymer-modified with PEN/ITO/SnO_2/PVK structure. Error bars represent the standard deviations from the adhesion strength results of five bonding materials.

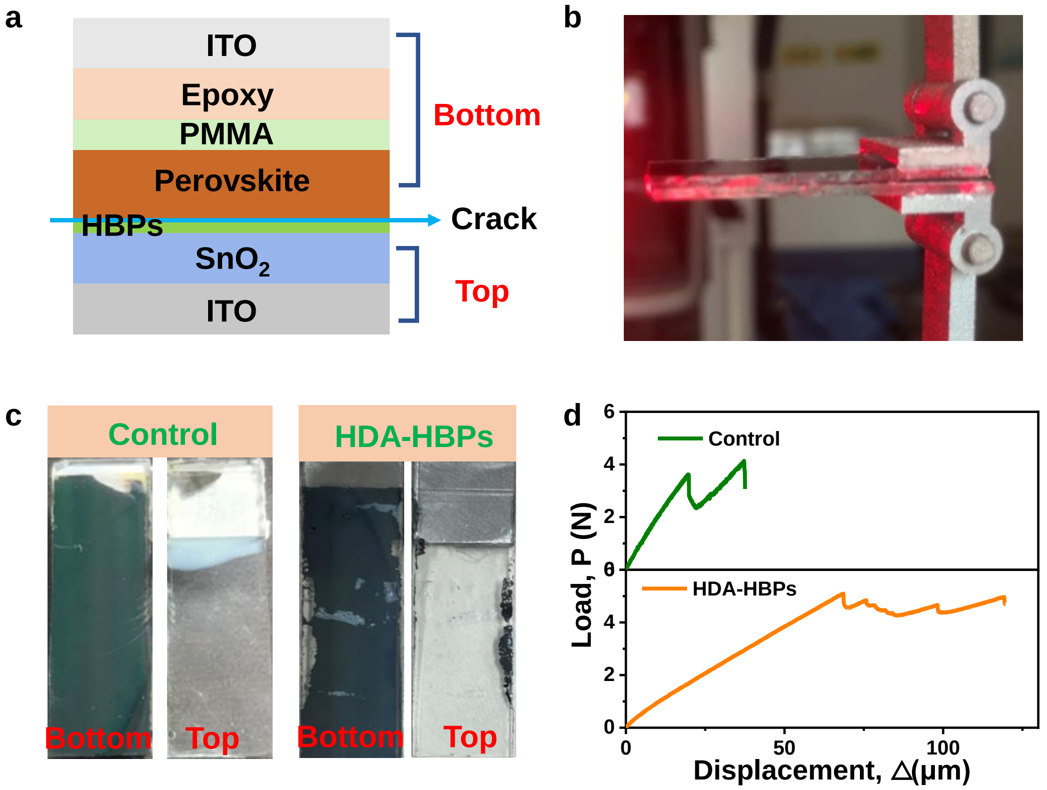

Supplementary Figure 30. (a) Schematic illustration of double cantilever beam (DCB) test. (b) Photograph of the DCB test equipment and (c) samples after DCB test. (d) Representative P-△ curves for the measurement of G_C of the "sandwich" DCB specimens with and without HDA-HBPs modification.

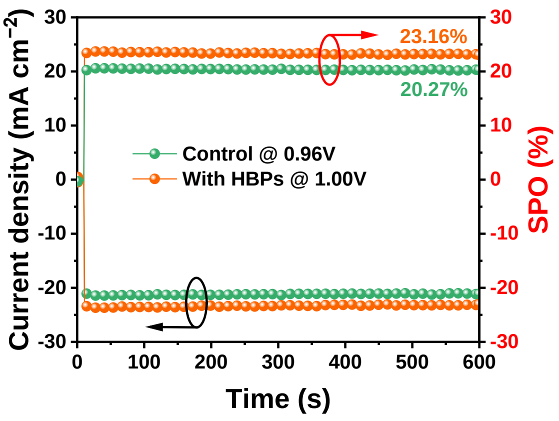

Supplementary Figure 31. Stabilized photocurrent and SPO for F-PSCs with and without HBPs modification.

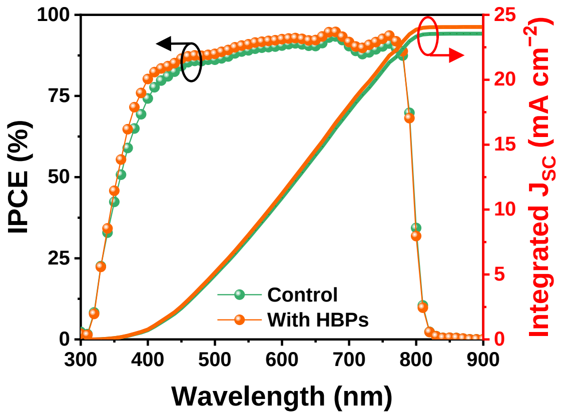

Supplementary Figure 32. External quantum efficiency (EQE) spectra and integrated current of the F-PSCs with and without HBPs modification.

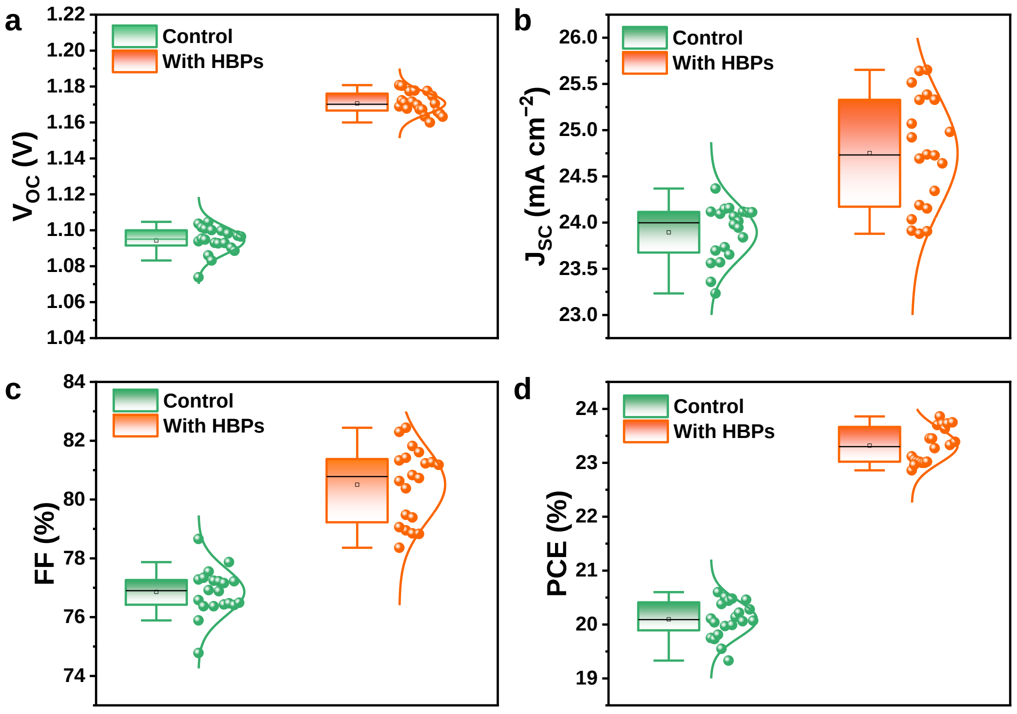

Supplementary Figure 33. (a) V_{OC}, (b) J_{SC}, (c) FF, and (d) PCE statistics of 20 flexible devices based on SnO_2 with and without HBPs modification. Error bars represent the standard deviations from the statistic results of two devices.

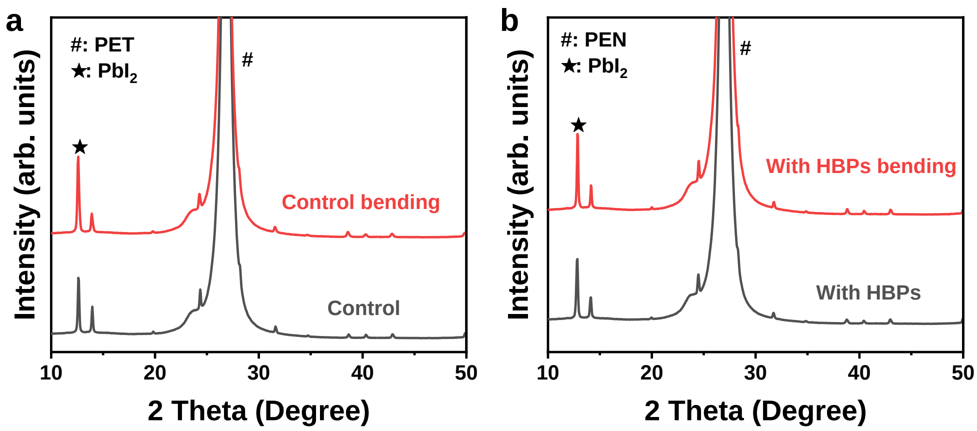

Supplementary Figure 34. XRD patterns of perovskite films deposited on flexible substrates based on (a) SnO_2 and (b) HBPs-modified SnO_2. (★: PbI_2; #: PET).

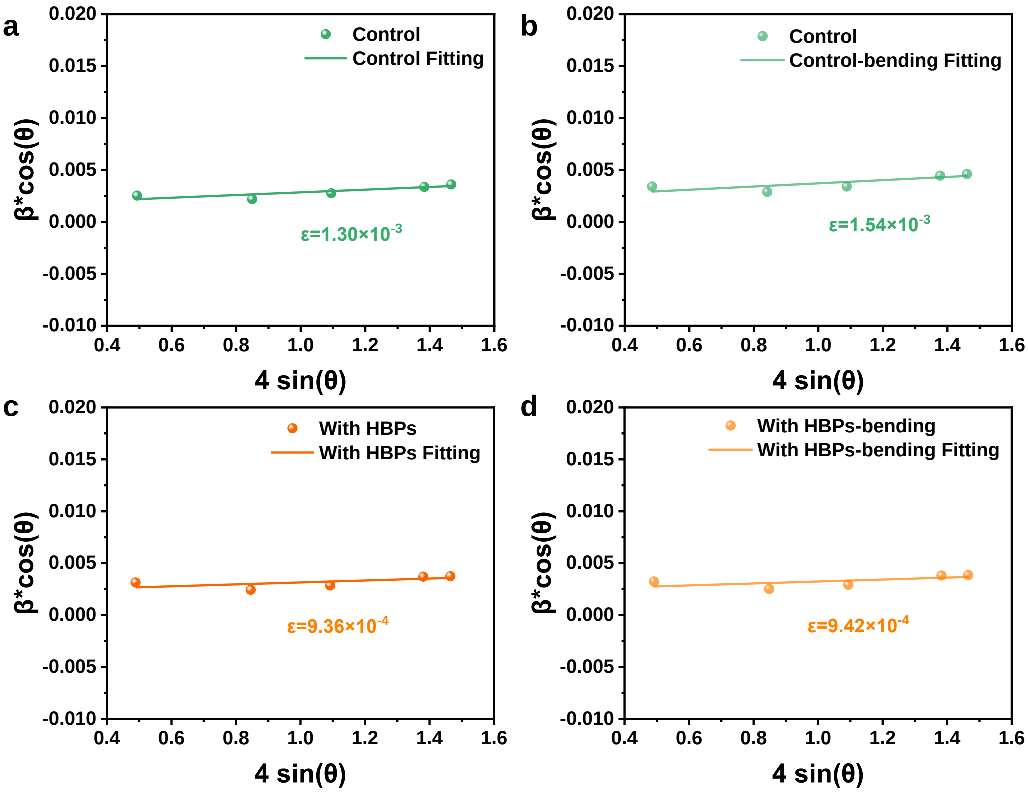

Supplementary Figure 35. Williamson-Hall plots of perovskite film deposited on (a,b) SnO_2 and (c,d) HBPs-modified SnO_2 before and after bending.

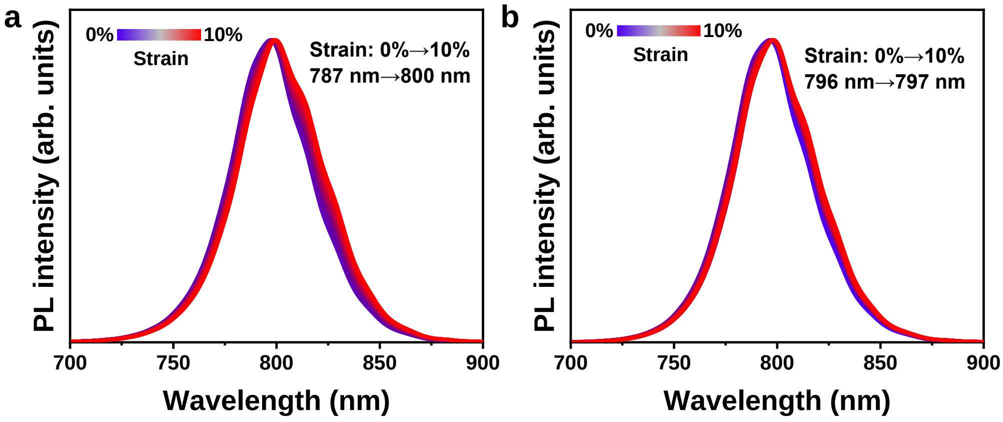

Supplementary Figure 36. Comparison of in situ PL spectra of perovskite films deposited on SnO_2 ETL without (a) and with (b) HBPs-modification as a function of strain from 0% to 10%.

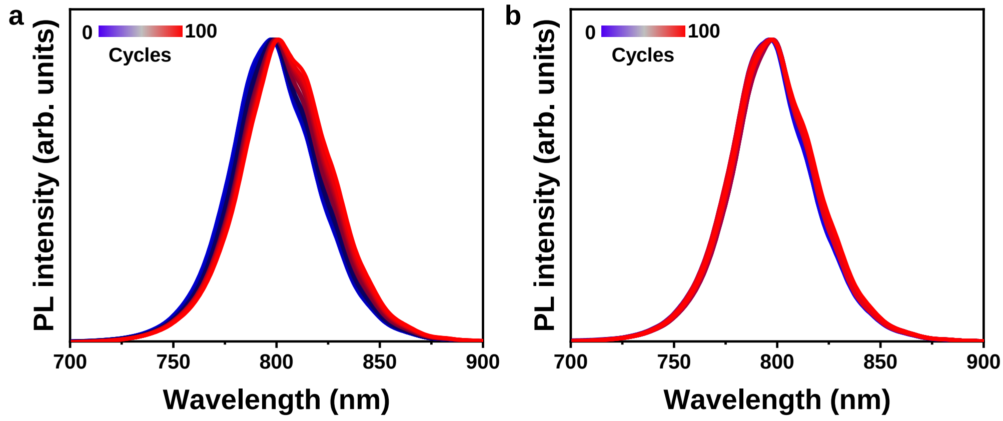

Supplementary Figure 37. Comparison of in situ PL spectra of perovskite films deposited on SnO_2 without (a) and with (b) HBPs-modified at 100 stretching cycles with the strain of 2%.

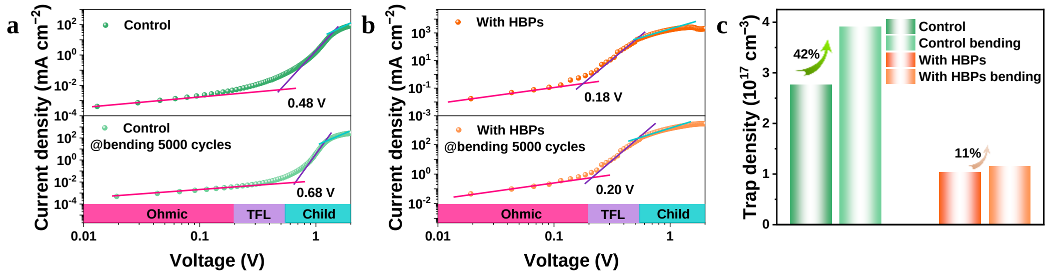

Supplementary Figure 38. SCLC measurements of perovskite films deposited on (a) SnO_2 and (b) HBPs-modified SnO_2 before and after bending with the electron-only

structures. (c) Comparison of trap density of control and HBPs-modified perovskite films before and after bending.

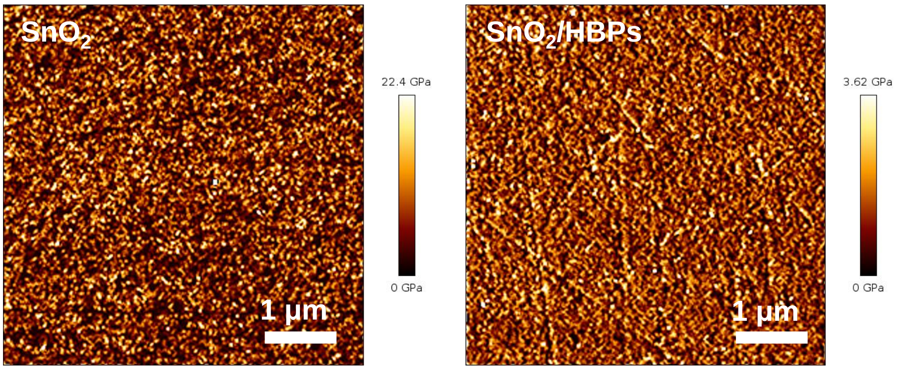

Supplementary Figure 39. Elastic modulus maps of SnO_2 and HBPs-modified SnO_2 films.

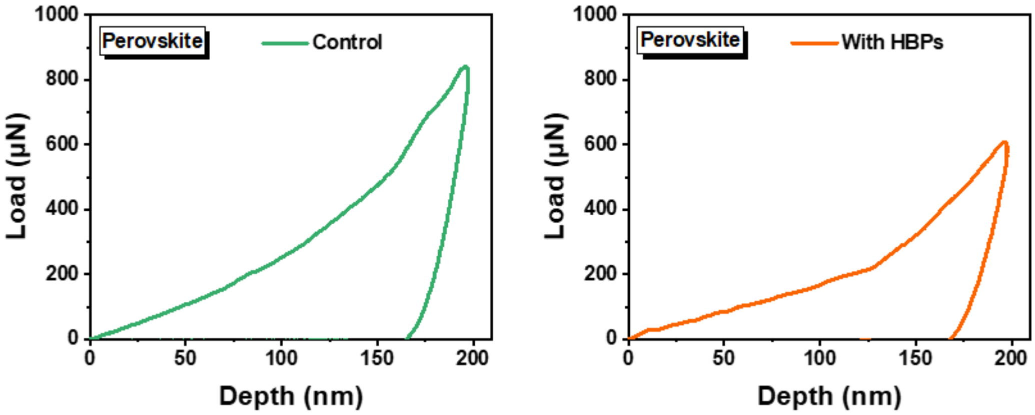

Supplementary Figure 40. Elastic modulus maps of perovskite films deposited on SnO_2 and HBPs-modified SnO_2 films.

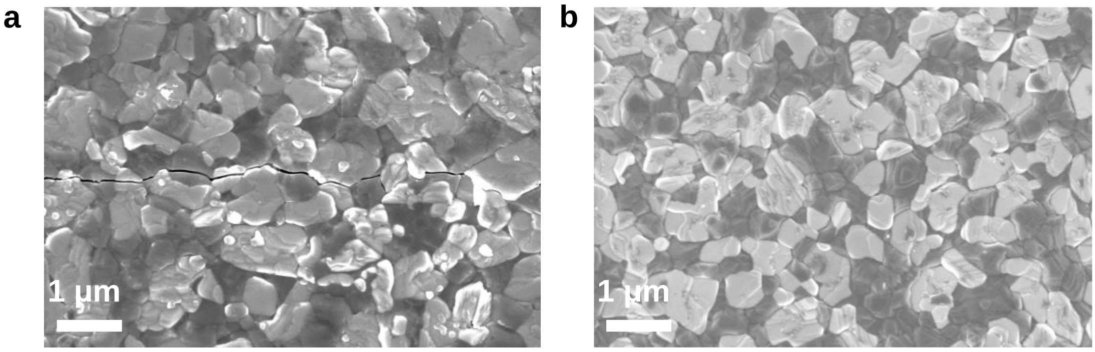

Supplementary Figure 41. The SEM images of perovskite films based on (a) SnO_2 and (b) HBPs-modified SnO_2 after bending.

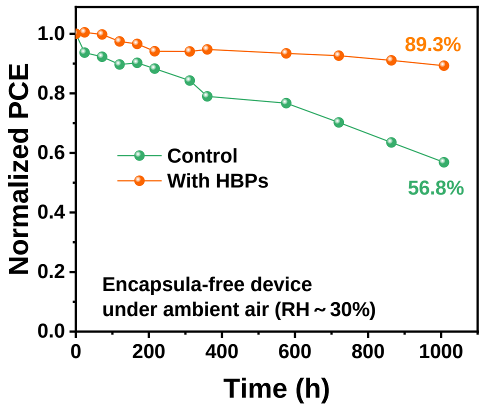

Supplementary Figure 42. Long-term stability of the encapsulation-free planar PSCs based on SnO_2 with and without HBPs modification under ambient air.

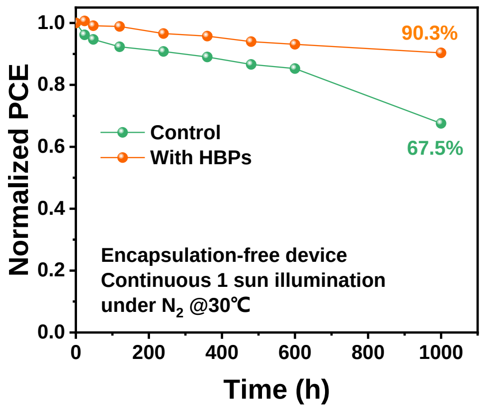

Supplementary Figure 43. Long-term stability of the encapsulation-free planar PSCs based on SnO_2 with and without HBPs modification under continuous 1-sun illumination in N_2 environment.

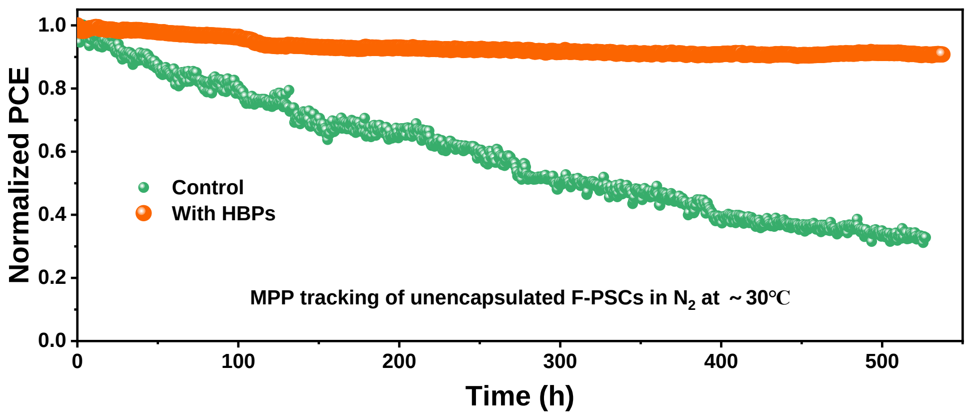

Supplementary Figure 44. The unencapsulated F-PSCs with and without HBPs modification under MPP tracking and one-sun illumination in N_2 at 30 °C

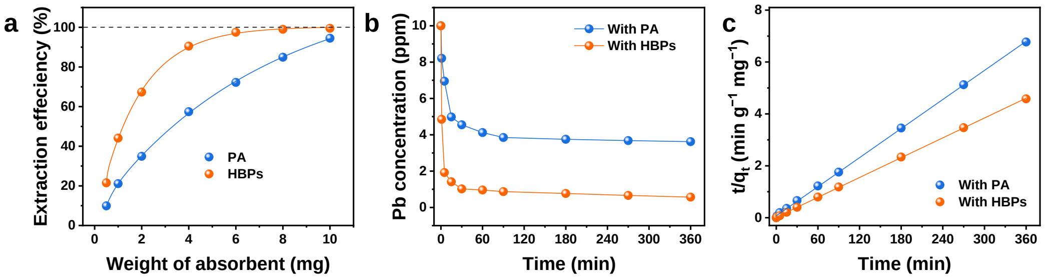

Supplementary Figure 45. Pb^{2+} absorption properties of different absorbents. (a) Influence of absorbent weight toward Pb^{2+} extraction efficiency. (b) Pb^{2+} sorption kinetics and the corresponding absorbed Pb^{2+} amount of PA and HBPs films. (c) The kinetics fitting curves of PA and HBPs films from a pseudo-second-order mode.

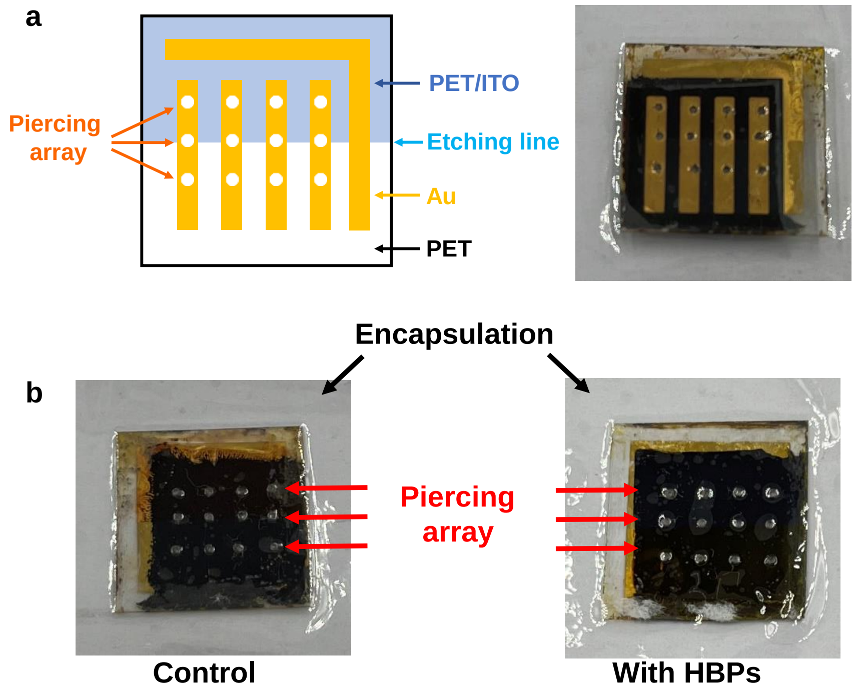

Supplementary Figure 46. (a) The schematics of damaged flexible PSCs piercing with a needle to form an array for lead leakage test and corresponding sample photo. (b) The flexible perovskite solar cells encapsulated by 10-μm-thick adhesive PET films.

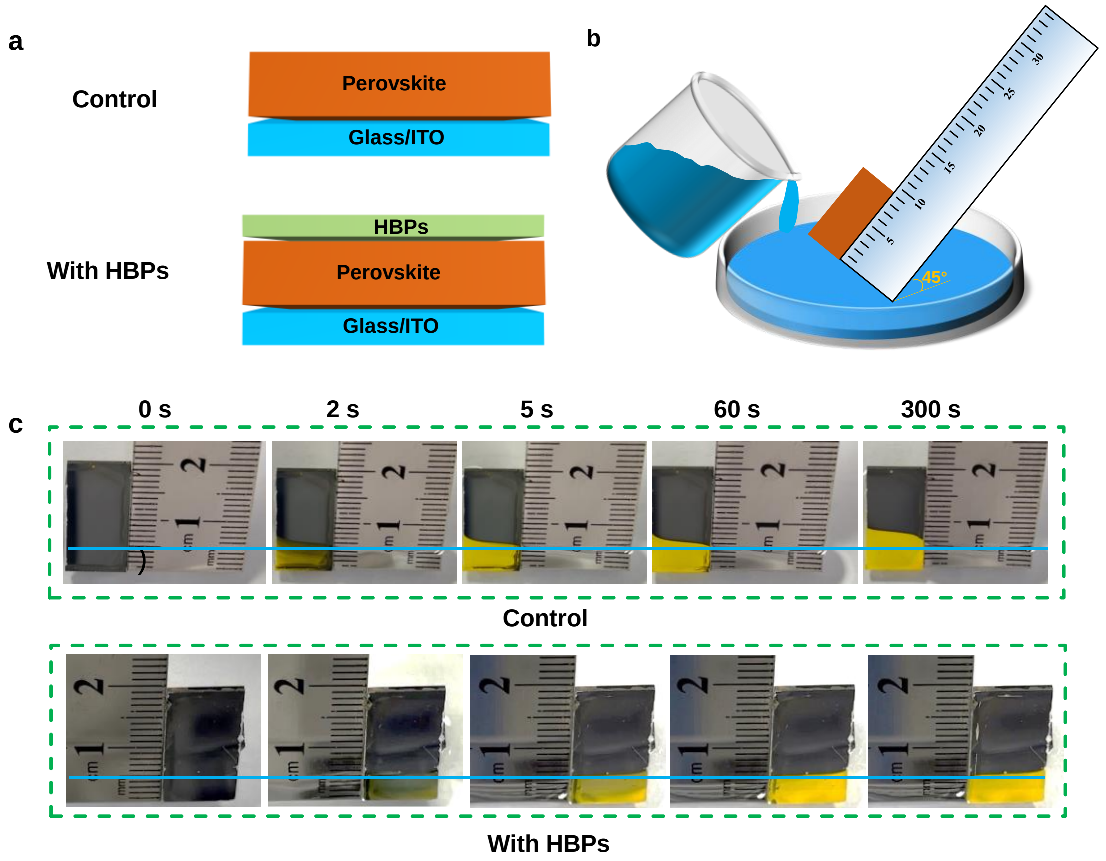

Supplementary Figure 47: (a) Sample of perovskite films with and without HBPs modification. (b) Schematic diagram of the degradation process of perovskite films. (c) Photos of the degradation evolution of perovskite films in aqueous solution.

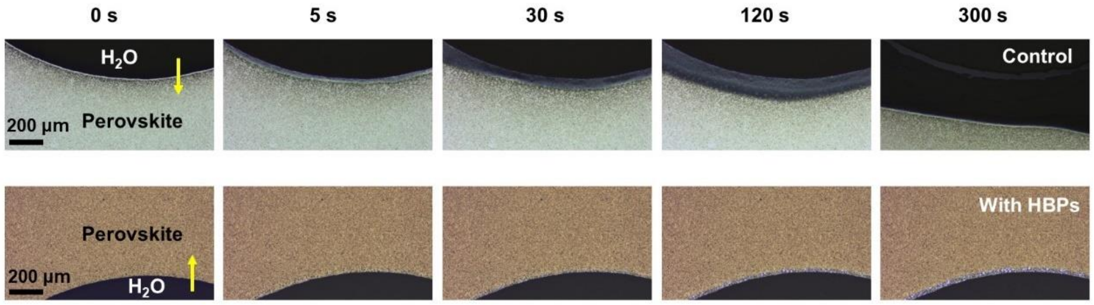

Supplementary Figure 48: Microscopic photos of the degradation process of perovskite films.

Supplementary Table 1. Corresponding binding energy position and relative areal intensity of the O 1*s* Peak.

	XPS peak parameters		O 1*s*	
Sample	SnO_2		HBPs-modified SnO_2	
Components	OH^-	O^{2-}	OH^-	O^{2-}
Binding Energy (eV)	532.2	530.7	531.9	530.7
Relative areal intensity	0.67	0.33	0.41	0.59

Supplementary Table 2. Statistical photovoltaic parameters of the devices based on SnO_2 with and without HBPs modification.

	V_{OC} (V)	J_{SC} (mA cm^{-2})	*FF*	PCE (%)
Control	1.117±0.014	23.99±0.48	76.52±1.49	20.50±0.51
HDA-HBPs	1.171±0.005	25.42±0.22	81.89±0.77	24.39±0.31

Supplementary Table 3. The fitting parameters of TRPLspectra in Fig. 3b.

ETLs	A_1 (%)	τ_1(ns)	A_2 (%)	τ_2(ns)	τ_{Ave}(ns)
Glass	41.7	122.6	58.3	758.9	239.8
SnO_2	16.4	18.1	83.6	393.9	89.4
SnO_2/HBPs-	21.2	3.9	78.8	31.7	12.7

Supplementary Table 4. The fitting parameters of TRPL spectra in Supplementary Figure 23.

ETLs	A_1 (%)	τ_1 (ns)	A_2 (%)	τ_2 (ns)	τ_{Ave} (ns)
SnO_2	30.1	49.1	69.9	314.9	119.6
SnO_2/HBPs	3.7	10.7	96.3	568.2	489.9

Supplementary Table 5. The energy levels information of SnO_2 and HBPs-modified SnO_2 films.

ETLs	E_g	E_l	E_{cutoff}	W_F	E_{Fermi}	E_{VB}	E_{CB}
SnO_2	3.92	3.56	16.61	4.61	-4.61	-8.17	-4.25
SnO_2/HBPs	3.93	3.02	16.24	4.98	-4.98	-8.00	-4.07

Supplementary Table 6. Statistical photovoltaic parameters of the flexible devices based on SnO_2 with and without HBPs modification.

	V_{OC} (V)	J_{SC} (mA cm^{-2})	FF (%)	PCE (%)
Control	1.094±0.007	23.89±0.30	76.85±0.80	20.10±0.34
With HBPs	1.170±0.005	24.75±0.60	80.50±1.5	23.31±0.32

Supplementary Table 7. The mechanical parameters of each layer for PSCs with and without HBPs modification.

Materials	Thickness (μm)	Young's modulus (Gpa)	Density (ρ, g cm^{-3})	Poisson's ratio
PET	50	2.37	1.38	0.41
SnO_2	0.04	5.414	6.95	0.29
SnO_2/HBPs	0.04	1.230	6.95	0.29
Control PVK	0.70	36.17	4.1	0.33
PVK with HBPs	0.70	27.05	4.1	0.33
Spiro-OMeTAD	0.06	20.98	1.4	0.3
Au	0.1	70	1.38	0.41

Supplementary Table 8. The sorption rate constant and adsorption capacity of PA and HBPs films.

Samples	K_2 (mg min^{-1} g^{-1})	q_e (mg g^{-1})
With PA	4.75	53.53
With HBPs	8.38	78.49

Supplementary Note 1.

Admittance spectroscopy (AS) and Mott−Schottky analysis[3, 4]

To reveal the passivation effect of the HBPs more directly and clearly, the admittance spectroscopy (AS) was conducted on the devices without and with the HBPs. AS is an effective technique for estimating both the energy level of trap states and the distribution of trap state density, which has been extensively applied to many photovoltaic systems, such as organic solar cells, Cu_2ZnSnS_4 solar cells, and PSCs. As the literature reported, for a p-type perovskite semiconductor, the defect activation energy (Ea) is approximately the depth of the trap state energy level (E_T) relative to the valence band maximum (VBM) energy level (E_{VBM}) of perovskite (Ea= $E_T - E_{VBM}$). Ea and the characteristic transition angular frequency (ω_0) can be expressed in the relation $\omega_0 = \beta T^2\, exp(-\frac{E_a}{k_B T})$, where β is a a temperature dependent parameter, T is the temperature and k_B is the Boltzmann's constant. The ω_0 is determined by the derivative of the capacitance−frequency spectrum. According to this equation, the Arrhenius plot ($ln(\frac{\omega_0}{T^2}) = ln\,\beta - \frac{E_a}{k_B T}$), and the value of Ea can be obtained from the slope of the Arrhenius plot line. The distribution of trap state density can be derived from the equation, $N_T(E_\omega) = -\frac{V_{bi}}{qW}\frac{dC}{d\omega}\frac{\omega}{k_B T}$, $E_\omega = k_B T\, ln(\frac{\omega_0}{\omega})$, where V_{bi} is the built-in potential, W is the depletion width, q is the elementary charge, C is the capacitance, and ω is the applied angular frequency. V_{bi} and W can be extracted from the Mott−Schottky analysis through the capacitance−voltage measurement. According to the depletion approximation, the C, V_{bi}, and W at the junction can be expressed in the relation, $\frac{C}{A} = \frac{\varepsilon\varepsilon_0 N}{W} = \sqrt{\frac{q\varepsilon\varepsilon_0 N}{2(V_{bi}-V)}}$, where A is active area, ε is the static permittivity of

perovskite, ε_0 is the permittivity of free space, N is the apparent doping profile in the depleted layer, and V is the applied bias. A Mott-Schottky plot ($\frac{A^2}{C^2} = \frac{2(V_{bi}-V)}{q\varepsilon\varepsilon_0 N}$) describes a straight line where the intersection on the bias axis determines V_{bi} and the slope gives the impurity doping density N. Then, the depletion width $W = \sqrt{\frac{2\varepsilon\varepsilon_0 V_{bi}}{qN}}$corresponding to the zero bias can be calculated.

Supplementary Note 2.

Mechanical Testing[5]

DCB specimens were loaded under displacement control in a universal testing machine (CMT 5305) from which a load, P, versus displacement, Δ, curve was recorded. The adhesive fracture energy, Gc (J/m^2), was measured in terms of the critical value of the applied strain energy release rate, G. Gc can be expressed in terms of the critical load, Pc, at which crack growth occurs, the crack length, a, the plane-strain elastic modulus, E, of the substrates and the specimen dimensions: width, B and half-thickness, h. Gc was calculated from Equation: $G_c = \frac{12Pc^2a^2}{B^2Eh^3}\left(1 + 0.64\frac{h}{a}\right)^2$. An estimate of the crack length was experimentally determined from a measurement of the elastic compliance, dΔ/dP, using the compliance relationship in Equation: $a = \left(\frac{d\Delta}{dP} * \frac{BEh^3}{8}\right)^{\frac{1}{3}} - 0.64h$. All Gc testing was carried out in laboratory air environment at ≈25 °C and ≈45% R.H. The specimen was loaded in tension with a displacement rate of 1 mm/min until reaching Pc to calculate dΔ/dP.

Supplementary Note 3.

Lead absorption property measurements

The lead content in the polluted water was determined via ICP-MS (Thermo Fisher Scientific). The amount of Pb adsorbed per unit mass (q_t) of film at the time (t) was determined by the expression $q_t = \frac{V(C_o - C_t)}{m}$, where C_o is the initial concentration, C_t is the concentration at time (t), V is the volume of the solution, and m is the net mass of the film. The adsorption kinetics were analyzed using a pseudo-second-order model: $q_t = \frac{tK_2 {q_e}^2}{1+tK_2 q_e}$, where q_e is the adsorption capacity (mg/g) of the adsorbent at equilibrium, K_2 (g min^{-1} mg^{-1}) is the equilibrium rate constant for the pseudo-second-order model. The experimental data plot of the pseudo-second-order kinetic model and which fitting (t/q_t versus t) of the kinetic adsorption results are shown in Figure S45.

Lead sequestration efficiency (SQE) was defined by $SQE(\%) = \left(1 - \frac{Pb\ leakage\ from\ control\ device}{Pb\ leakage\ from\ HBPs\text{−}modified\ device}\right) \times 100\%$

Supplementary References

1. Wang, H. et al. Room-temperature autonomous self-healing glassy polymers with hyperbranched structure. *Proc. Natl. Acad. Sci. U.S.A.* **117,** 11299-11305 (2020).
2. Jiang, Q. et al. Surface passivation of perovskite film for efficient solar cells. *Nat. Photonics* **13,** 460-466 (2019).
3. Duan, H.-S. et al. The identification and characterization of defect states in hybrid organic–inorganic perovskite photovoltaics. *Phys. Chem. Chem. Phys.* **17,** 112-116 (2015).
4. Ye, S. et al. A Breakthrough Efficiency of 19.9% Obtained in Inverted Perovskite Solar Cells by Using an Efficient Trap State Passivator Cu(thiourea)I. *J. Am. Chem. Soc.* **139,** 7504-7512 (2017).
5. Rolston, N. et al. Effect of Cation Composition on the Mechanical Stability of Perovskite Solar Cells. *Adv. Energy Mater.* **8,** 1702116 (2018).
