# Supplementary material for: Hyperbranched polymer functionalized flexible perovskite solar cells with mechanical robustness and reduced lead leakage
Source: Nat Commun. 2023 Oct 13;14:6451. doi: 10.1038/s41467-023-41931-1 (PMC10576085; doi:10.1038/s41467-023-41931-1)
Supplement: Supplementary file 3 — Description of Additional Supplementary Files [file 41467_2023_41931_MOESM3_ESM.pdf]

File name: Supplementary Movie 1
Description: Process and instrument of the fatigue testing of the flexible PSCs.
